# Diode laser transscleral cyclophotocoagulation causes intraocular collamer lens displacement in pseudophakic eye: a case report

**DOI:** 10.1186/s12886-021-02026-x

**Published:** 2021-06-29

**Authors:** Wei Wei, Xueqing Yu, Lu Yang, Chan Xiong, Xu Zhang

**Affiliations:** Affiliated Eye Hospital of Nanchang University, Jiangxi Research Institute of Ophthalmology and Visual Science, Jiangxi Clinical Research Center of Ophthalmic Disease, Nanchang, Jiangxi People’s Republic of China

**Keywords:** Glaucoma; uveitis, Intraocular collamer lens, Transscleral cyclophotocoagulation, Case report

## Abstract

**Background:**

With the rapid development of intraocular collamer lens (ICL) operation, it is foreseeable that we will encounter a large number of glaucoma patients with ICL implantation history. However, our current understanding of the treatment of glaucoma patients with ICL is limited. Hence we report a rare case of refractory glaucoma after intraocular collamer lens and intraocular lens implantation in a patient who underwent unsuccessful transscleral cyclophotocoagulation, which led to intraocular collamer lens displacement, angle closure and uncontrolled intraocular pressure.

**Case presentation:**

A 39-year-old woman presented with intractably elevated intraocular pressure in the right eye. Since her intraocular collamer lens implantation surgery in 2017, her intraocular pressure had remained over 40 mmHg while using 3 types of anti-glaucoma medications. The patient had a history of phacoemulsification and posterior chamber phakic intraocular lens implantation for complicated cataracts secondary to uveitis in 2006. On gonioscope examination, there were signs of pigment dispersion, and the anterior chamber angle was open. Ultrasound biomicroscopy examination showed contact and rubbing between the intraocular collamer lens and posterior surface of the iris. And typical advanced glaucomatous optic neuropathy and visual field defects were observed. Transscleral cyclophotocoagulation was performed to control the intraocular pressure and prevent further visual field loss. However, the intraocular collamer lens was displaced after transscleral cyclophotocoagulation, which resulted in formation of a shallow anterior chamber 1 week later, angle closure and loss of intraocular pressure control 1 month later, even though the maximum dose of anti-glaucoma medication was used. Finally, an Ahmed glaucoma valve was successfully implanted in her anterior chamber, and the glaucoma was controlled, as observed at the 10-month follow-up.

**Conclusions:**

Pigment dispersion is a common phenomenon after intraocular collamer lens implantation and may accelerate the progression of glaucoma. Transscleral cyclophotocoagulation should be carefully considered in glaucoma patients with elevated intraocular pressure after intraocular collamer lens implantation, given that transscleral cyclophotocoagulation may cause intraocular collamer lens displacement.

## Background

Intraocular collamer lens (ICL) implantation has become a preferred surgical treatment for patients with ametropia, although our understanding of its postoperative complications is still insufficient. Pigment dispersion is a common complication after ICL implantation and may result in pigmentary glaucoma. However, it has not been reported whether pigment dispersion affects glaucoma progression in patients who have a history of uveitis and ocular hypertension without glaucomatous optic neuropathy (GON). Transscleral cyclophotocoagulation (TSCP) is an effective treatment for refractory glaucoma, is easy to perform and is noninvasive. However, the efficacy and safety of TSCP in glaucoma patients with both ICLs and intraocular lenses (IOLs) remain to be investigated. Hence, we report a case of complex glaucoma in a patient with two IOLs following TSCP, which caused ICL displacement, angle closure and uncontrolled intraocular pressure (IOP).

## Case presentation

A 39-year-old woman was referred to the glaucoma clinic for management of elevated intraocular pressure and vision loss in the right eye after ICL implantation (V4c Visian ICL, Staar Inc., Monrovia, CA) in 2017. Her preoperative examinations showed high myopia in the right eye and no abnormalities in the left eye. Before ICL implantation, ultrasound biomicroscopy (UBM) revealed a deep anterior chamber, open angles and correct IOL position in the right eye. No glaucomatous optic disc change was observed in her right eye. After ICL implantation, her IOP fluctuated between 40 and 55 mmHg. She had a history of phacoemulsification and posterior chamber phakic IOL implantation due to complicated cataracts secondary to uveitis in 2006. Regular ophthalmological re-examinations were conducted after IOL implantation, and temporarily elevated IOP of 29 mmHg was observed in 2016. Her IOP returned to normal after treatment with anti-glaucoma medication (the specific medication is unknown), followed by irregular medication. She had no history of trauma or systemic disease. A family history of glaucoma or high myopia was denied.

**Fig. 1:**
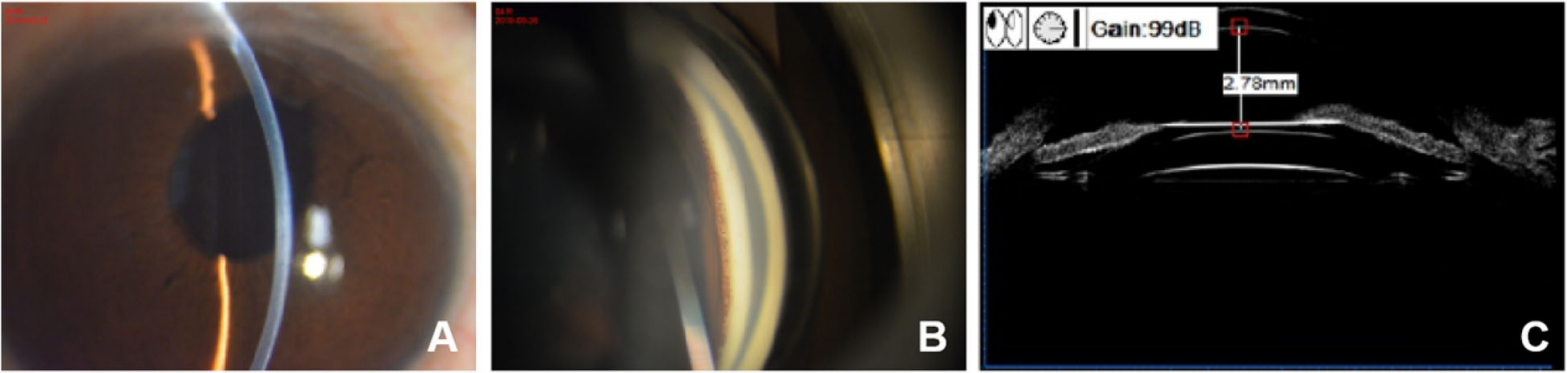
Right eye shows deep anterior chamber depth and the aqueous fluid is clear (**A**). Gonioscopy reveals the angle is open and pigmentation (Grade II in Scheie’s pigmentation grading) on the trabecular meshwork (**B**). UBM examination shows contact and rubbing between ICL and posterior surface of the iris (C)

On admission examination in the glaucoma clinic, the patient was found to have an IOP of 43 mmHg while using 3 types of anti-glaucoma medications, and her best-corrected visual acuity was 20/20 in the right eye. Slit-lamp examination showed that both the ICL and IOL were well centred, and a few pigmented keratic precipitates were present on the corneal endothelium with pigment deposition on the surface of the ICL, although the aqueous fluid was clear (Fig. 1a). Gonioscopy revealed that the whole angle was open and that there was pigmentation on the trabecular meshwork (Fig. 1b). UBM examination showed contact and rubbing between the ICL and posterior surface of the iris (Fig. 1c). The cup to disc ratio was 0.8. The Humphrey visual field test (24–2 SITA-standard) indicated typical advanced visual field defects (mean defect = − 24.13 dB *P*<0.5%) that were consistent with GON on optical coherence tomography. No evidence of pigment dispersion or GON was observed in the left eye.

**Fig. 2:**
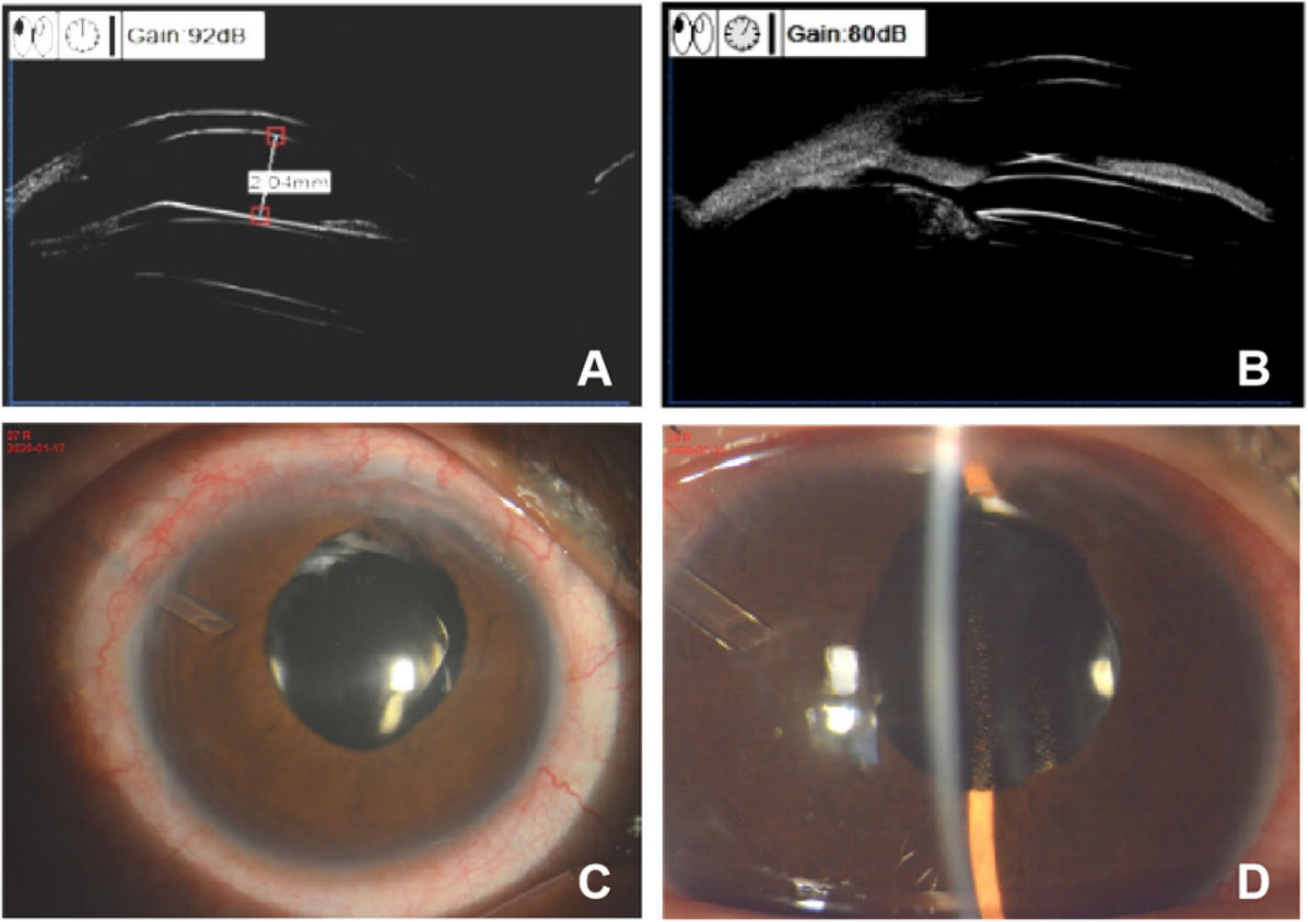
After TSCP, UBM shows uneven depth of the anterior chamber and the ICL move forward in one week (**A**), and 1/2 of the angle close in one month (**B**). The Ahmed glaucoma valve is well positioned in her right eye 10 month after operation (**C**), the anterior chamber is narrow and there is pigmentation on the ICL and IOL surfaces (**D**)

The patient was diagnosed with refractory glaucoma, and her IOP was controlled to 25–35 mmHg by oral methazolamide and an intravenous drip of mannitol. Then, she received diode laser TSCP, which was performed with 20 spots (2000 mW) at 2 s each, sparing the temporal quadrant to avoid vascular damage. Postoperative medications included anti-inflammatory, anti-infection and 3 types of anti-glaucoma drugs. Her IOP was 18 mmHg the day after surgery, and a physical examination showed no obvious abnormality in the right eye, but the BCVA had decreased to 20/40. One week later, the anterior chamber became shallow, and the ICL was incarcerated in the pupil. UBM showed uneven depth of the anterior chamber, and the ICL had moved forward (Fig. 2a). The patient’s postoperative IOP increased to 30 mmHg over 1 month while using the maximum dose of anti-glaucoma medication, and 1/2 of the angle was closed on UBM (Fig. 2b) and gonioscopy. An Ahmed glaucoma valve (AGV) model FP7 (New World Medical, Rancho Cucamonga, CA) was implanted 2 months after TSCP without moving the IOLs (Fig. 2c, Fig. 2d). Her IOP was well controlled without anti-glaucoma medication, her visual acuity remained 20/40, and no progression of glaucoma occurred in the next 10 months.

## Discussion and conclusions

Pigment dispersion (PD) is a common complication after ICL implantation [1] and may lead to secondary glaucoma [2] Ye et al. reported a case of pigment dispersion glaucoma secondary to ICL implantation due to direct contact of the phakic ICL with the posterior iris [2]. Although pigment dispersion glaucoma secondary to ICL surgery has been described several times, there are no reports concerning patients who have a history of uveitis and ocular hypertension undergoing ICL surgery. In our case, the patient showed PD and rapid progression of glaucoma after the ICL operation. Inadequate posterior chamber space after ICL implantation and chronic uveitis due to contact between the ICL and iris were two possible reasons for PD [3]. Considering the possibility of PD after ICL surgery, gonioscopy and UBM should be routinely used for postoperative follow-up, especially in patients with a history of uveitis, to detect PD early and intervene in glaucoma in a timely manner.

TSCP, as an effective treatment for reducing IOP, is easy-to-operate, noninvasive and normally used to treat refractory glaucoma [4] Liu et al. described a patient who developed uveitis-glaucoma-hyphema syndrome following vitrectomy and IOL implantation and was successfully treated by TSCP. This case suggests that TSCP can be a treatment option when the IOL is slightly tilted [5]. However, in our patient, TSCP failed 1 month after surgery. After TSCP, the patient’s visual acuity was partially lost, and IOP was only transiently controlled. The ICL was displaced and caused the formation of a shallow anterior chamber within 1 week, angle closure and loss of IOP control within 1 month, although the maximum dose of anti-glaucoma medication was used. This result suggested that TSCP in the used fashion of 20 spots with 2 s and 2000 mW may not be a good choice for patients with good visual acuity. In addition, the location of the IOL and ICL needs to be considered carefully. In this case, we performed TSCP in the superior quadrant, nasal quadrant and inferior quadrant, which made the ciliary body shrink in three quadrants, and then the ICL fixed in the ciliary sulcus was incarcerated in the remaining quadrant. In addition, an intense diode laser may cause photovaporization, and the exploding effect may lead to ICL displacement. Thus, the range and diode power of the laser should be considered preoperatively when performing TSCP in patients with both an IOL and ICL. Furthermore, it would seem necessary to avoid the direction of pupil displacement.

There is no significant difference between TSCP and the AGV placement in the success rate of neovascular glaucoma (NVG) treatment. Choy et al. noted that NVG eyes with AGV implants were more likely to lose vision and develop complications than those treated with TSCP [6]. However, our case shows that the AGV may be a better choice for patients with refractory glaucoma and an IOL and ICL. Given that TSCP may cause ICL shifting, which affects the patient’s refractive status and vision, cases should be carefully re-evaluated before selecting treatment for glaucoma patients with ICLs. The AGV has been proven safe and effective in reducing IOP in patients with refractory glaucoma [7]. Since the AGV is in the majority of cases implanted in the anterior chamber, it has less effect on the posterior chamber space and was more suitable for this case. Meanwhile, we hypothesize that AGVs can remove pigments from their tubes and alleviate PD progression.

In conclusion, we present a case of refractory secondary glaucoma in a patient with an ICL and IOL who underwent an unsuccessful TSCP, and the IOP was controlled by AGV implantation. Through this tortuous course of treatment, we obtained 3 valuable tips. First, ICLs should be carefully implanted in eyes with a history of uveitis, and the eye should be checked for PD. Then, TSCP should not be indiscriminately performed on eyes with an ICL, and AGV might be an alternative in treatment of refractory glaucoma. Finally, since every operation has specific complications, comprehensive follow-up after surgery is particularly important.

## Data Availability

Not applicable.

## Abbreviations

ICL: Intraocular collamer lens
GON: Glaucomatous optic neuropathy
TSCP: Transscleral cyclophotocoagulation
IOL: Intraocular lens
IOP: Intraocular pressure
UBM: Ultrasound biomicroscopy
AGV: Ahmed glaucoma valve
PD: Pigment dispersion
NVG: eovascular glaucoma
